# *HER2* mutations in Chinese patients with non-small cell lung cancer

**DOI:** 10.18632/oncotarget.11313

**Published:** 2016-08-16

**Authors:** Zhengbo Song, Xinmin Yu, Zhiyong Shi, Jun Zhao, Yiping Zhang

**Affiliations:** ^1^ Department of Medical Oncology, Zhejiang Cancer Hospital, Hangzhou, China; ^2^ Key Laboratory Diagnosis and Treatment Technology on Thoracic Oncology, Hangzhou, China

**Keywords:** non-small cell lung cancer, HER2 mutation, prevalence, genetic variability, treatment

## Abstract

**Background:**

*ERBB2* (*HER2*) is a driver gene identified in non-small cell lung cancer (NSCLC). The prevalence, clinicopathology, genetic variability and treatment of *HER2*-positive NSCLC in Chinese population are unclear.

**Patients and Methods:**

Eight hundred and fifty-nine patients with pathologically confirmed NSCLC were screened for *HER2* mutations using Sanger sequencing. Next-generation sequencing (NGS) was performed in positive cases. *HER2* amplification was detected with FISH. Overall survival (OS) was evaluated using Kaplan-Meier methods and compared with log-rank tests.

**Results:**

Twenty-one cases carrying *HER2* mutations were identified with a prevalence of 2.4%. *HER2* mutations were more frequently encountered in females, non-smokers and adenocarcinoma. NGS was performed in 19 out of 21 patients, The results showed 16 cases with additional genetic aberrations, most commonly associated with *TP53* (*n* = 6), followed by *EGFR* (*n* = 3), NF1 (*n* = 3), *KRAS* (*n* = 2) and other mutations. One patient harbored *HER2* amplification. Four patients with stage IV received afatinib treatment, and three showed stable disease with a median progression-free survival of 4 months and one patient was diagnosed with progressive disease.

**Conclusion:**

*HER2* mutations represent a distinct subset of NSCLC. NGS showed that *HER2* mutations commonly co-existed with other driver genes. Afatinib treatment displayed moderate efficacy in patients with HER2 mutations.

## INTRODUCTION

Lung cancer is one of the leading causes of cancer-related death worldwide [1]. Testing for driver genes in patients with non-small cell lung cancer (NSCLC) is the new standard of clinical decision-making [2-3]. Targeted therapies focusing on driver genes increase the quality of life and prolong survival [4-7].

*ERBB2* (*HER2*) is expressed in solid carcinomas including cancers of the breast, stomach, lung and pancreas [8]. Preclinical and clinical studies have confirmed that *HER2* is a driver gene in NSCLC [9-12]. Three principal mechanisms of *HER2* alteration include: protein overexpression, gene amplification and gene mutations [13]. In NSCLC, HER2 mutations were identified to represent a distinct subset of driver genes that usually excluded with other common driver genes like EGFR, KRAS and ALK, based on published studies. [14-16].

Despite the relative rarity of HER2 mutations, several studies have reported *HER2* mutations in NSCLC [17-21]. However, most studies focused on the prevalence and clinical characteristics. The prognosis, treatment and especially genetic variability of HER2 have not yet to be well investigated.

In the present study, we investigated the frequency of *HER2* mutations in a large cohort of Chinese NSCLC, along with the treatment and prognosis. We determined genetic variability with next-generation sequencing.

## RESULTS

### Patient characteristics

Totally, eight hundred and fifty-nine patients with pathologically confirmed NSCLC were screened for HER2 mutations. Twenty-one patients were identified carrying HER2 mutations. Among the 21 patients, 14 were females and seven of males. The patients' median age was 60 years (range, 39-70). Four patients were ever or current smokers and 17 without smoking history. The majority of patients presented with adenocarcinoma (*n* = 19), and the remaining two patients were diagnosed with squamous cell carcinoma. All the stages were represented at diagnosis: four patients with stage I, three patients with stage II, seven patients with stage III, and seven patients with stage IV disease.HER2 mutations occurred at a significantly higher frequency in female, never-smokers with adenocarcinoma when compared with *HER2* negative patients. The clinicopathological characteristics of present study are listed in Table 1.

**Table 1:** Clinical characteristics comparison in patients with and without HER2 mutations

Characteristics	HER2 positive	HER2 negative	*P*
Gender			
Male	7	493	0.019
Female	14	345	
Age			0.198
<60	10	514	
≥60	11	322	
Smoking status			0.004
Never	17	412	
Former/current	4	426	
Histology			0.095
Adenocarcinoma	19	624	
Squamous cell cancer	2	214	
Stage at diagnosis			0.825
I-IIIA	14	539	
IIIB/IV	7	299	

### Gene analysis

**Figure 1:**
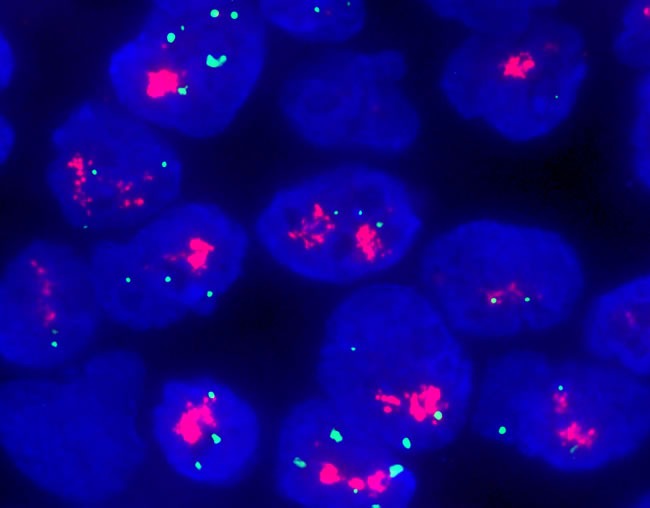
*HER2* amplification in a patient (NO. 12) with a HER/CEP17 ratio of 7.3 by FISH detection.

All the mutations were in-frame insertions of exons 20 and 19 resulting in duplication of amino acids YVMA associated with codon YVMA 776-779 ins and two other codons (one with VC 777-778 *ins* and the other with GSP 781-783ins). *HER2* amplification was detected in one patient (No.12) with *HER*/*CEP17* of 7.3 by FISH (Table 2 and Figure 1).

**Table 2:** Clinical characteristics of patients carrying a HER2 mutation in their tumors (*n* = 21)

Case	Gender/age	Histology subtype	HER2 mutation subtype	Co-current genes	First-line chemotherapy	efficacy	PFS/months	EGFR-TKI	efficacy	PFS/month	OS/month
1	M/69	Enteric	VC 777-778 ins	KRAS	-	-	-	-	-	-	60+
2	F/41	Lepidic pred	YVMA 776-779 ins	No detection	Pemetrexed/carboplatin	PR	5.5	-	-	-	55+
3	F/58	Solid pred	GSP 781-783ins	TP53	Gemcitabine/cisplatin	SD	4.6	Afatinib	SD	5.5	21
4	F/65	Solid pred	YVMA 776-779 ins	EGFR	Pemetrexed/carboplatin	PD	1.2	Icotinib	PD	1.2	12.5
5	F/58	Papillary pred	YVMA 776-779 ins	TP53	-	-	-	-	-	-	56+
6	M/65	Papillary pred	YVMA 776-779 ins	NF1+TP53	Gemcitabine/cisplatin	SD	4.8	Icotinib	PD	1.1	18
7	F/55	Papillary pred	YVMA 776-779 ins	No detection	-	-	-	-	--	-	45
8	F/50	Micropapillary pred	YVMA 776-779 ins	EGFR	Pemetrexed/cisplatin	PR	5.0	Icotinib	PR	14.4	17.7
9	M/39	AIS	YVMA 776-779 ins	-	-	-	-	-	-	-	39
10	F/70	Papillary pred	YVMA 776-779 ins	-	Pemetrexed/carboplatin	SD	2.8	Afatinib	SD	3.5	14.5
11	F/55	Papillary pred	YVMA 776-779 ins	ROS1	-	-	-	-	-	-	48+
12	F/41	Solid pred	YVMA 776-779 ins	HER2 amplification	paclitaxel/carboplatin	SD	4.6	-	-	-	58+
13	F/69	Acinar pred	YVMA 776-779 ins	EGFR+TP53	Gemcitabine/cisplatin	PD	1.0	Icotinib	PD	1.0	6.5
14	M/62	Minimally invasive	YVMA 776-779 ins	KRAS	Gemcitabine/cisplatin	SD	4.2	Icotinib	PD	1.2	12
15	F/54	Acinar pred	YVMA 776-779 ins	BRCA1	Gemcitabine/cisplatin	PR	8.5	Gefitinib	PD	2.0	24
16	F/64	Papillary pred	YVMA 776-779 ins	CDKN2A+ARID1A	-	-	-	-	-	-	58+
17	F/63	Papillary pred	YVMA 776-779 ins	NF1+DDR2+TP53	Gemcitabine/cisplatin	PR	10.0	-	-	-	45
18	M/60	Micropapillary pred	YVMA 776-779 ins	-	-	-	-	Afatinib	SD	4.0	45
19	F/55	Squamous cell	YVMA 776-779 ins	NF1+TP53	Gemcitabine/carboplatin	SD	2.2	-	-	-	36+
20	M/64	Squamous cell	YVMA 776-779 ins	EXT1,SMARCA4	Gemcitabine/cisplatin	PR	5.0	Erlotinib	SD	2.2	54
21	M/66	Papillary pred	YVMA 776-779 ins	KRAS	Docetaxel/platinum	PD	1.0	Afatinib	PD	1.0	13

Abbreviations:Pred: predominant; Adenocarcinoma in situ:AIS;Stable disease:SD;Progression disease:PD;partial response: PR.

The tumors of 19 patients were further analyzed by NGS. Concurrent driver genes were detected in 16 patients: six carrying *TP53* mutation, three harboring *EGFR* mutations(two with exon19 delete and one with exon21 L858R mutation), three carrying *NF1* mutations, and two patients with *KRAS* mutation. Additional gene aberrations are listed in Table 2.

### Treatment

Among the 859 patients, 511 had a postoperative onset of recurrence or metastasis and 423 died. Of the 21 patients with HER2-positve, fourteen with advanced stage or recurrent NSCLC received palliative chemotherapy or targeted treatment. Eleven patients were treated with EGFR-TKIs including seven with first-generation agents (erlotinib, gefitinib or icotinib) and four with the second-generation inhibitor (afatinib). In the seven patients treated with first-generation EGFR-TKIs, the median PFS was 2.0 months. Notably, three patients were treated for concurrent *HER2* and *EGFR* mutations, and only one patient responded to first-generation EGFR-TKI treatment.

Four patients received afatinib monotherapy. Disease was controlled in three of these patients with a median progression-free survival of 4.0 months. One patient showed progressive disease with afatinib treatment.

All of the 14 patients received first-line chemotherapy. The median PFS of first-line treatment was 4.6 months. No PFS difference of first-line chemotherapy existed between

Patients with and without HER2 mutations in their tumors (4.6 *vs*.5.2 months, *P* = 0.45).

### Survival analysis

**Figure 2:**
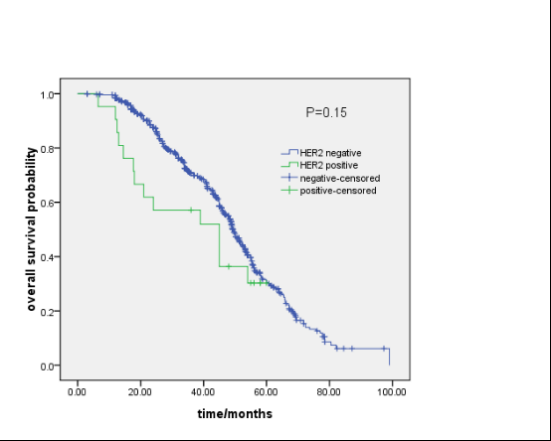
Survival analysis: *HER2-*positive *vs* *HER2-*negative patients (49.3 months *vs*.45.0 months, *P* = 0.150).

Of the 859 patients, 62 were lost to follow-up. None of the 21 patients with a HER2 mutations in their tumor were lost to follow-up. No differences in age, histology or stage were seen between *HER2*-positive and HER2-negative patients. The median overall survival in the current study was 49.1 months. *HER2*-negative patients presented with a tendency for longer survival than *HER2*-positive patients (49.3 months *vs*.45.0 months, *P* = 0.150)(Figure 2).

**Table 3:** Clinical efficacy of afatinib in HER2 mutation NSCLC patients

Author	Number	Male/female	Efficacy	PFS	OS
Mazie'res et al.[23]	3	NP	SD:2/PR:1	NP	NP
De Grève et al.[24]	3	0/3	SD:2/PR:1	4M	14M
Current study	4	2/2	SD:3/PD:1	3.5M	17.75M

Abbreviations: NP:not reported; SD: stable disease;PR:partial response;PD:progression disease;M:month

## DISCUSSION

We evaluated the prevalence, prognosis and genetic variability of *HER2* mutations in a large cohort of lung cancers in Asian population. Our results demonstrated that patients with a HER2 mutation in their tumor were responsive to *HER2* inhibitor treatment. *HER2* mutations frequently encountered with other driver genes, which may influence therapeutic decision-making.

The prevalence of HER2 mutations has been reported previously to range from 1% to 6% in NSCLC [16-22]. Wang and colleagues reported 1943 NSCLC and found a *HER2* mutation rate of 1.3%. Eleven of 204 NSCLC patients harbored *HER2* mutations by Shan et al. study [20]. Based on previous studies, the presence of *HER2* mutations was associated with female, never smokers and lung adenocarcinoma. In the present study, *HER2* mutations were associated with never-smoker status and female patients, which is consistent with previous studies [16-22]. Notably, two patients with HER2 mutations were confirmed with squamous cell carcinoma in our cohort. To our knowledge, our report of the two cases involving squamous cell carcinoma with a *HER2* mutation is the first of its kind.

In previous studies, *HER2* amplification was not routinely detected. Hence, no data of concurrent amplification and mutation were reported. In the present study, *HER2* amplification was detected in one patient using FISH. The results suggested that the two molecular alterations were not commonly associated.

Due to the rarity of *HER2* mutations in NSCLC, no study investigated genes co-existing with these mutations. In current report, NGS was used to detect the *HER2* mutation and concurrent genes. We found that 16 of 19 patients with a *HER2* mutation in their tumors carried concurrent genes. Notably, three of our concurrent *HER2* and *EGFR-*positive patients were treated with first-generation EGFR-TKI inhibitors (two with exon19 delete and one with exon21 L858R mutation), and two presented with progressive disease. The limited number of patients in the current study suggested the poor efficacy of first-generation EGFR-TKIs against *HER2* mutations. In these patients, second-generation EGFR-TKIs such as afatinib and dacomtinib may be a better option.

Evidence supporting *HER2*-targeted therapy in NSCLC is still scarce. In the largest published series, Mazieres et al. reported that 16 patients with *HER2* mutation were treated resulting in a median PFS of 5.1 months [23]. The anti-HER2 therapies included trastuzumab, afatinib and lapatinib. Interestingly, three patients were treated with afatinib resulting in 100% disease control. De Grève et al. reported that three NSCLC patients with *HER2* mutation treated with afatinib showed objective response [24]. We report disease control in three out of four patients with *HER2* mutations treated with afatinib. Based on these two studies and our data (Table 3), we conclude that afatinib monotherapy is appropriate for patients with *HER2* mutations. In addition, recent data have demonstrated that afatinib plus paclitaxel may be particularly more effective than single afatinib treatment, hence, combinitaion afatinib and chemotherapy may be a better option in HER2-positive NSCLC patients [25].

Due to the rarity of the molecular subtypes, the survival of *HER2* mutations in NSCLC was not well studied. In one study by Tomizawa et al., a tendency of shorter overall survival in *HER2* mutations was reported compared with *HER2-*negative cases [26]. In another cohort, the overall survival of *HER2* mutations did not differ significantly from other cohorts carrying *EGFR*, *ALK*, *KRAS*, and *BRAF* mutations [10]. In the present cohort, no survival differences were found in patients with and without *HER2* mutations. However, there was a trend toward longer survival in *HER2*-negative patients. The results may due to the imbalance in treatment after recurrence or metastasis.

Our study limitations are related to its retrospective design with only 21 patients with *HER2* mutation. Further, only four patients were treated with afatinib, suggesting caution in interpreting afatinib efficacy in *HER2* mutants.

Our report suggests that the disease with *HER2* mutations represents a subset of NSCLC. We suggest that *HER2* mutations are not exclusive to other genetic aberrations detected by NGS. We also found that afatinib is a potentially novel treatment option for this subgroup of patients.

## MATERIALS AND METHODS

### Patient selection

A total of 859 patients with pathologically-confirmed NSCLC at Zhejiang Cancer Hospital from January 2008 through December 2014, were retrospectively enrolled in this study. The inclusion criteria were: pathologically confirmed diagnosis of NSCLC, availability of samples for *HER2* analysis, and absence of chemotherapy or radiotherapy before pathological diagnosis. Histology was based on the 2004 World Health Organization classification. Lung cancer staging was performed in all patients according to the 7^th^ TNM classification. This study was approved by the Review Board of the Zhejiang Cancer Hospital, Zhejiang, China.

### *HER2* mutation analysis

A microscopy was used to ensure the tumor tissues analyzed had more than 20% tumor contents. Genomic DNA was extracted using the QIAamp DNA Tissue kit (Qiagen,Germany) following the manufacturer's standard protocol. *HER2* exon 20 mutation analysis was carried out using Sanger sequencing. Briefly, the entire coding region of HER2 exon 20 was amplified using the forward primer, 5′-GCCATGGCTGTGGTTTGTGATGG-3′ and reverse primer,5′-ATCCTAGCCCCTTGTGGACATAGG-3.The Refseq accession number for *HER2* gene analyzed in this study is NM_001289937.

### *HER2* FISH

The Vysis PathVysion HER2 DNA Probe Kit (Abbott Laboratories) was used for *HER* amplification according to the standard manufacturer's protocol. At least 40 cells were analyzed in each case by two pathologists and classified according to the 2013 ASCO/CAP evaluation systems for *HER2* amplification in breast cancer: *HER2*/*CEP17* ratio ≥ 2.0 or < 2.0, average *HER2* copy number ≥ 6.0 signals/cell.

### NGS detection

The *HER2* samples were subjected to targeted next generation sequencing (NGS). DNA was extracted by digestion in proteinase K buffer, followed by purification with DNeasy Tissue kit (Qiagen) with some modifications. The 416 cancer-related genes were captured using a 5′-biotinylated probe. Deep sequencing was performed with IlluminaHiSeq4000 using PE75 V1 Kit (Illumina, San Diego, USA). Cluster generation and sequencing was performed according to the manufacturer's protocol. Results were visualized in the Integrative Genomics Viewer (IGV) and manually analyzed. A 1% cutoff for variant calls was used. The median sequencing coverage was > 800. A comprehensive protocol of NGS is provided as a supplementary file.

### Efficacy evaluation

Tumor evaluation during afatinib treatment was performed every four to eight weeks, or earlier if significant signs of progression were observed. Objective tumor responses were evaluated based on the Response Evaluation Criteria in Solid Tumors (RECIST 1.1).

### Statistical analysis

The relationship between *HER2* mutation and clinicopathological variables was analyzed using the chi-square test. Survival curves were calculated using the Kaplan-Meier method starting with NSCLC diagnosis until death or last follow-up. Progression-free survival (PFS) of EGFR-TKI was defined as the time from therapy to documented progression or death due to any cause. Statistical analysis was performed with the SPSS 16 software (Chicago, IL, US). *P* < 0.05 was considered statistically significant. The median follow-up period was 55 months (5-75) and the last follow-up was conducted on July 31, 2015.
